# Conditional VAE for personalized neurofeedback in cognitive training

**DOI:** 10.1371/journal.pone.0335364

**Published:** 2025-10-31

**Authors:** Imad Eddine Tibermacine, Samuele Russo, Gianmarco Scarano, Giancarlo Tedesco, Abdelaziz Rabehi, Amel Ali Alhussan, Doaa Sami Khafaga, Marwa M. Eid, El-Sayed M. El-kenawy, Christian Napoli

**Affiliations:** 1 Department of Computer, Control and Management Engineering, Sapienza University of Rome, Rome, Italy; 2 Department of Psychology, Sapienza University of Rome, Rome, Italy; 3 Laboratory of Telecommunications and Smart Systems, Faculty of Sciences and Technologies, University of Djelfa, Djelfa, Algeria; 4 Department of Computer Sciences, College of Computer and Information Sciences, Princess Nourah bint Abdulrahman University, Riyadh, Saudi Arabia; 5 Faculty of Artificial Intelligence, Delta University for Science and Technology, Mansoura, Egypt; 6 Jadara Research Center, Jadara University, Irbid, Jordan; 7 School of ICT, Faculty of Engineering, Design and Information and Communications Technology (EDICT), Bahrain Polytechnic, Isa Town, Bahrain; 8 Applied Science Research Center, Applied Science Private University, Amman, Jordan; 9 Institute for Systems Analysis and Computer Science, Italian National Research Council, Rome, Italy; 10 Department of Computational Intelligence, Czestochowa University of Technology, Czestochowa, Poland; Commonwealth Scientific and Industrial Research Organisation, AUSTRALIA

## Abstract

Machine learning (ML) offers great potential in healthcare, especially in the analysis of complex physiological signals like electroencephalography (EEG). EEG recordings hold valuable insights into neurological function and can aid in diagnosing various conditions. In this work, we explore the use of a Conditional Variational Autoencoder (CVAE) that injects a binary health label (healthy or orthopedic impairment) into both the encoder input and the latent space coupled with the extracted features, leveraging the conditional input vector to learn representations specific to different health conditions. Our study involved using two public OpenNeuro datasets [1,2]. From the healthy dataset we randomly selected seven subjects to match the seven impaired participants; in both sets we retained the same 11 scalp channels (C3, Cz, C4, FC3, FCz, FC4, CP3, CPz, CP4, F3, F4). Six descriptors-Short time Fourier Transform (STFT), Hurst Exponent (HE), Detrended Fluctuation Analysis (DFA), Correlation Dimension (CD), Kolmogorov-Sinai Entropy (permutation entropy; KS-proxy), and the Largest Lyapunov Exponent (LLE)-are extracted channel-wise and concatenated to form the input feature vector, which distills distinct characteristics from the EEG signals. We rigorously evaluated the performance of our CVAE model in combination with each feature extraction technique. The *conditional* supply of class labels to both encoder and decoder enabled the CVAE to achieve 93% accuracy on the unseen test split of the dataset with precision of 93%, a recall of 93%, and an F1-score of 0.93 outperforming re-trained CNN baselines. These results highlight the promise of CVAEs and the significance of well-suited feature extraction for robust EEG classification. This work could contribute to the development of automated healthcare diagnostic tools.

## 1 Introduction

EEG provides a non-invasive method to observe the complex electrical activity of the brain, establishing itself as a fundamental tool in diagnosing and studying various neurological conditions such as epilepsy, sleep disorders, and cognitive impairments [3]. Traditional EEG analysis relies heavily on the subjective interpretation by clinicians, but the advent of machine learning techniques, particularly through brain-computer interfaces (BCI), has transformed this process [4,5]. BCIs leverage EEG signals to facilitate interaction between humans and digital or robotic devices, enhancing not only medical diagnostics but also the quality of life for individuals with cognitive impairments through technologies such as gesture control and eye tracking [6].

The primary functionality of BCIs is predicated on the accurate detection of specific brain activities that translate into computer commands [6,7]. This application is prominently executed using EEG due to its non-invasive advantages, where users perform Motor Imagery (MI) tasks [8,9] to generate recognizable EEG patterns [7,10]. MI involves imagining motor movements without actual execution, activating specific cerebral cortex regions and leading to changes in EEG rhythm signals [11], such as event-related desynchronization (ERD) and synchronization (ERS) across mu and beta wave spectra [12,13].

However, EEG signals are inherently noisy and non-stationary, with significant variability between individuals due to physiological differences [14–16]. This variability presents a considerable challenge in processing EEG signals, requiring effective pre-processing to improve signal quality and robust feature extraction methods [17,18]. The transition from traditional BCI methodologies to more advanced implementations integrates not only handling the inherent noise and variability in EEG data, but also improving the interpretability and usability of EEG-based commands in real-world applications [19–21].

Deep learning models, particularly CVAEs, have emerged as powerful tools in this context, especially in the realm of EEG classification within BCIs [22,23]. CVAEs excel in learning complex high-dimensional representations from EEG data, facilitating both the classification and reconstruction of EEG signals [9,24]. Using CVAEs, it is possible to improve the accuracy and efficiency of BCIs, allowing a more precise interpretation of user intentions from EEG signals [25,26]. The ability of CVAEs to handle both classification and reconstruction tasks provides valuable insight into the underlying patterns of brain activity, pushing the limits of what can be achieved with EEG data in both medical and technological applications [27–29].

EEG signals have been extensively studied using a wide array of analytical methods rooted in information theory [30], nonlinear dynamics [31], fractal theory [32], and complex systems techniques [33–36]. The inherently non-linear nature of EEG signals makes them particularly suitable for analysis using nonlinear dynamics methods, including techniques such as Hurst exponents [37], Lyapunov exponent [38], entropy measures [39], and fractal dimensions [40]. These methods have been instrumental in distinguishing between EEG signals from individuals with neurological conditions such as epileptic seizures and those from healthy individuals [41,42].

The potential of machine learning in healthcare care is transformative, especially in the analysis of complex physiological signals such as EEG, which holds significant promise for the diagnosis and understanding of neurological conditions. Traditional machine learning techniques, such as Support Vector Machines (SVMs) [43,44] and decision trees [45], have shown early success in EEG classification, demonstrating the capabilities of automated analysis. However, the complex and high-dimensional nature of EEG signals poses challenges that often exceed the capabilities of traditional ML approaches, driving the adoption of more powerful deep learning models [28].

Feature extraction is critical in EEG analysis as it distills complex and noisy signals into representations that highlight salient characteristics suitable for machine learning models [46]. Various techniques contribute to this process: The Hurst Exponent quantifies long-range dependencies in time series, providing information on the persistence of EEG signal, while the detrended fluctuation analysis measures scaling properties and fractal-like patterns, revealing the underlying dynamics [47,48]. The correlation dimension assesses the complexity and non-linear dynamics of brain signals, and the Kolmogorov-Sinai entropy estimates the complexity and irregularity of the signal, reflecting potential differences in the brain activity states. The Short-time Fourier Transform analysis decomposes a signal into overlapping segments analyzed using the Fourier transform, providing a time-frequency distribution that elucidates the evolving spectral characteristics [49,50].

Deep learning, particularly through the use of Convolutional Neural Networks (CNNs) [51] and Recurrent Neural Networks (RNNs), has outperformed traditional machine learning techniques in various EEG classification tasks [52,53]. The impressive capabilities of Deep Convolutional Neural Networks (DCNNs) [54] in EEG-based attention decoding tasks demonstrate their ability to analyze complex EEG signals and extract meaningful patterns related to attention, speech perception, and noise suppression. These networks are specially designed to process the complex spatial-temporal patterns characteristic of multi-channel EEG recordings [55,56]. To address the challenges of limited datasets, data augmentation using CVAEs has been shown to improve model performance significantly [57].

Variational Autoencoders (VAEs) are potent in feature extraction from EEG data, capable of modeling complex distributions and uncovering underlying neurological patterns that may indicate subtle differences related to conditions such as obesity [58,59]. The integration of CVAEs in our EEG analysis framework is based on established encoder-decoder principles. The encoder component of a CVAE transforms the complex, high-dimensional EEG input into a compact latent representation, which captures essential patterns relevant to the classification task [60]. By conditioning the CVAE on health status labels (‘Healthy’ or ‘Unhealthy’), the model learns to discriminate between these conditions effectively. The decoder then attempts to reconstruct the original EEG signal from the latent representation, allowing the model to address dual objectives: minimizing reconstruction loss to ensure accurate signal regeneration and enhancing classification accuracy by producing features that distinguish between different EEG patterns [61,62]. The integration of EEG with advanced deep learning techniques through BCIs represents a significant advancement in neurotechnology [63,64]. This synergy not only enhances diagnostic capabilities and interaction modalities in medical and technological fields but also paves the way for future innovations in understanding and interfacing with human brain dynamics [65,66].

However, the success of Deep Learning models hinges on the quality of the input data. Raw EEG signals often contain noise and complex dynamics, making direct classification challenging. Feature extraction techniques play a pivotal role in transforming raw EEG signals into meaningful representations that highlight relevant characteristics, thus improving model performance.

This paper explores the synergy between various feature extraction methods and a CVAE model for robust EEG classification. Our primary research questions include:

1. **Feature Optimization:** Which feature extraction method, among the Hurst Exponent, DFA, CD, STFT, Kolmogorov-Sinai Entropy, and their combinations, yields the most informative representations for EEG classification?
2. **CVAE Reconstruction:** To what extent can this novel model accurately reconstruct EEG signals, and how does this ability relate to its classification performance?
3. **CVAE Performances:** How does the CVAE’s performance compare to existing state-of-the-art approaches for EEG classification?

Methodologically, the present investigation utilizes EEG datasets that concentrate on motor imagery tasks, employing sophisticated feature extraction techniques such as the Hurst Exponent, Detrended Fluctuation Analysis, Short-time Fourier Transform, Correlation Dimension, and Kolmogorov-Sinai Entropy. Subsequently, we train a Conditional Variational Autoencoder, which is predicated on both the extracted EEG features and health condition labels, to proficiently model and classify the underlying neural patterns. A comprehensive preprocessing pipeline guarantees data consistency and reliability, encompassing noise elimination, signal filtration, and data normalization to improve the quality of the subsequent analysis.

This study constitutes the first motor-imagery EEG framework that embeds a binary health-condition label *simultaneously* in the encoder input and in the latent code of a conditional variational auto-encoder trained on a physiologically grounded, hand-crafted feature stack. This dual conditioning unifies interpretable signal descriptors with modern generative modeling, producing a compact representation that surpasses current discriminative pipelines and establishes a new benchmark for diagnosis-oriented EEG analysis.

The principal contribution of our research resides in illustrating the CVAE’s capacity to acquire meaningful and discriminative representations from EEG signals, in addition to its potential for precise signal reconstruction. These outcomes not only enhance our comprehension of how CVAEs can be optimally employed for EEG analysis but also establish a new standard for the automated diagnosis of neurological disorders utilizing EEG data. Our endeavor creates a significant reference framework that may inform the future advancement of EEG-based diagnostic tools, thereby improving their accuracy and applicability within clinical settings.

## 2 Materials and methods

### 2.1 Dataset

This study leverages two public EEG-MI datasets with distinct cohorts and acquisition setups. We analyze only EEG signals (ignoring auxiliary modalities), convert all recordings to a common montage, and harmonize sampling and preprocessing across datasets.

- **Healthy dataset [2].** EEG Motor Movement/Imagery: 109 volunteers, 64-channel 10–10 montage, original sampling 160 Hz (EDF+). Each subject performed multiple MI runs comprising left/right hand, both fists, and feet tasks (executed and imagined). We used only MI runs (baselines discarded).
- **Orthopedic-impairment dataset [1].** Multimodal MI in patients with orthopedic impairment: 7 participants, EEG montage with 18 channels, original sampling 500 Hz, MI trials recorded in multiple sessions. We use only the EEG stream for analysis.

**Subject selection and class definition:** To achieve parity in participant counts, we selected 7 subjects from the healthy dataset to match the impaired cohort; where age/sex metadata were available, we preferentially chose healthy subjects to approximate the clinical cohort’s demographics. We define a binary label y∈{0,1} for *healthy* (0) vs. *orthopedic impairment* (1). All per-window labels inherit the subject’s group label (task identity is used only as an auxiliary input where stated elsewhere).

**Common montage:** According to the channel-description files, both datasets share an identical subset of 11 electrodes: C3, Cz, C4, FC3, FCz, FC4, CP3, CPz, CP4, F3, F4. All subsequent analyses are confined to this montage; no interpolation or channel dropping is required.

**Signal harmonization:** All recordings are re-referenced as in their source datasets, notch-filtered, and band-pass filtered from 8–50 Hz using a zero-phase, fourth-order Butterworth design (filtfilt). Signals are then resampled to *f*_*s*_ = 125 Hz with zero-phase FIR decimation so that each 1 s epoch contains exactly 125 samples. To ensure consistency across datasets, only channels in the common montage are retained before resampling.

**Segmentation and event handling:** Following filtering, continuous recordings are segmented into **non-overlapping 1 s windows** (125 samples). For the healthy dataset we exclude baseline (eyes-open/closed) runs and retain only MI runs; for the impaired dataset we retain all MI trials. Trial/event markers are used solely to isolate MI segments; we do not stratify by MI task for the main binary classification unless otherwise stated.

**Quality control and exclusion:** We remove windows with missing event annotations and windows with non-physiological amplitudes (>|100|μV after re-referencing). Remaining windows are z-scored per recording (mean and standard deviation computed from training data only to avoid leakage).

**Balancing windows across groups:** After selecting 7+7 subjects, residual imbalances in the number of MI windows across groups and subjects are addressed in two steps: (i) per-subject stratified undersampling to equalize window counts across subjects within each group; and (ii) if a residual class imbalance <5% remains, we apply inverse-frequency weights in the classification loss,


$$\omega_{c}\;=\;\frac{N}{2\;N_{c}}\;,\;c\in \{\text{healthy},\text{impaired}\},$$

where *N* is the total number of training windows and *N*_*c*_ is the number from class *c*. In practice, after step (i) the classes are near-balanced and weights remain close to 1; we do *not* use extreme weights (the earlier placeholder $\omega_{\text{impaired}}\;=\;214$ is removed as a typographical artifact).

The final analysis set contains an equal number of participants per group (7 healthy, 7 impaired), a standardized 11-channel montage, and uniformly preprocessed EEG at 125 Hz segmented into 1 s windows. Dataset-specific acquisition details and accession information are given in [1,2]; our evaluation protocol (stratified 80/20 split and metrics) is described later.

**Covariates sensitivity:** EEG differs between individuals for reasons that are not related to the disease status, including age- and sex-related effects on rhythm power, peak frequencies, and long-range dependence. The two public datasets used here were curated independently and provide incomplete demographic metadata; consequently, a formal covariate-controlled analysis (e.g., regression or matched subsampling) cannot be reported without introducing unverifiable assumptions or discarding large portions of data.

Several design choices in the present study reduce—but do not eliminate—covariate influence: (i) a fixed stratified 80/20 split (subject proportions preserved) maintains class balance but does not enforce subject exclusivity; (ii) per-recording *z*-scoring removes absolute amplitude differences that can correlate with age/sex; (iii) a fixed 11-channel common montage avoids spatial-coverage disparities; and (iv) participant counts are balanced across cohorts. These steps improve comparability at the signal and sampling levels, yet unmeasured covariates (age, sex, medication, sleep, cap fit) may still contribute residual variance. Reported group differences should therefore be interpreted as condition-linked patterns within the recorded cohorts, not as causal effects independent of demographics. A covariate-adjusted evaluation requires complete metadata and is outside the scope of the current re-analysis of public datasets.

### 2.2 Preprocessing and enhancements

#### 2.2.1 Filtering:

Raw EEG was notch-filtered at the *dataset-appropriate* mains frequency (50 or 60 Hz), then band-pass filtered between 8 and 50 Hz using a zero-phase, fourth-order Butterworth design (filtfilt). To suppress edge transients, signals were mirrored by 3 s at both ends before zero-phase application. The upper cut-off preserves *γ*-band activity (30–50 Hz), which indexes motor preparation and sensorimotor integration during motor-imagery tasks [67,68], while attenuating higher-frequency myogenic artifacts. (Resampling to 125 Hz follows the dataset harmonization in Sect 2.1; anti-aliasing low-pass was applied prior to decimation.)

#### 2.2.2 Segmentation:

Following filtering, recordings were partitioned into **non-overlapping 1 s windows** (125 samples at $f_{s}\;=\;125$ Hz). Windows were drawn *only* from MI intervals indicated by event markers; baseline/rest periods were excluded. Any window that straddled an event boundary (onset/offset) was discarded to avoid label contamination. The 1 s duration is a canonical compromise for MI decoding and for estimating long-range dependency measures (Hurst, DFA) on short segments; preliminary tests with 0.5 s and 2 s windows yielded no consistent gains, while 1 s aligns with the temporal resolution of the *γ*-band STFT features used downstream.

#### 2.2.3 Channel montage:

All recordings were restricted to the 11 common electrodes listed earlier (C3, Cz, C4, FC3, FCz, FC4, CP3, CPz, CP4, F3, F4); no interpolation or channel exclusion was necessary, ensuring an identical spatial representation across datasets. Re-referencing followed the convention of each source dataset prior to reduction to the common montage.

#### 2.2.4 Normalization:

Each channel was 𝒵-scored on a per-recording basis (mean and standard deviation computed from the *training split* only and then applied to validation/test windows of the same recording) to remove inter-subject amplitude variability and prevent train–test leakage. Normalization precedes all feature computations.

### 2.3 Feature extraction

All features are computed *per 1 s window* and *per channel* on *z*-scored data (mean and standard deviation estimated on the training split only to avoid leakage). Unless stated otherwise, logarithms are natural and constants are fixed across all experiments.

**Short-Time Fourier Transform (STFT) band power:** Each 1 s window has *N* = 125 samples at *f*_*s*_ = 125 Hz. We compute the magnitude STFT with a *Hann* window of length *N*_*w*_ = 64 samples and 75% overlap (hop *h* = 16), yielding $M=\lfloor \frac{N-N_{w}}{h}\rfloor \;+\;1=4$ frames per window. The DFT size is $N_{fft}=128$ (zero-padding as needed). Let *X*(*k*,*m*) be the STFT at bin *k* and frame *m*, with bin frequency $f_{k}=\frac{kf_{s}}{N_{fft}}$. Define band index sets


$$B_{\alpha }=\{k:\;8\leq f_{k}<13\},\;B_{\beta }=\{k:\;13\leq f_{k}<30\},\;B_{\gamma }=\{k:\;30\leq f_{k}\leq 50\}.$$

Log-power per band uses a stabilizer $\epsilon =10^{-8}$:


$${BP}_{B}\;=\;\frac{1}{M}\sum_{m=1}^{M}\;\sum_{k\in B}\;\log\;(|X(k,m){|}^{2}+\epsilon ),\;B\in \{\alpha ,\beta ,\gamma \}.$$

**Hurst exponent ($\hat{H}$, rescaled range):** For scales s∈{8,16,32,64,125} (50% overlap), we compute the classical rescaled-range statistic. Given a demeaned segment $\{x_{t}{\}}_{t=1}^{s}$, form the cumulative deviate series $Y(u)=\sum_{t=1}^{u}x_{t}$ (u=1,…,s); the range is $R(s)=\max_{u}Y(u)\;-\;\min_{u}Y(u)$ and the standard deviation $S(s)=\sqrt{\frac{1}{s-1}\sum_{t=1}^{s}x_{t}^{2}}$. The segment statistic is R(s)/S(s). Averaging over the segments for each *s*, we fit


$$\log 𝔼[R(s)/S(s)]\;=\;a+\hat{H}\log s$$

via OLS; the shortest scale is omitted when fewer than two cycles of the dominant rhythm fall within *s*.

**Detrended Fluctuation Analysis (DFA):** For each 1 s window, build the integrated profile $Y(k)=\sum_{t=1}^{k}(x_{t}\;-\;\bar{x})$, partition into ⌊N/s⌋ non-overlapping segments of length s∈{8,16,32,64,125}, fit and subtract a least-squares linear trend *Y*_*s*_(*k*) within each segment, then compute


$$F(s)\;=\;\sqrt{\frac{1}{N}\sum_{k=1}^{N}(Y(k)-Y_{s}(k){)}^{2}}.$$

The DFA exponent $\alpha_{DFA}$ is the slope of logF(s) vs. logs (OLS on valid scales).

**KolmEn (permutation/ordinal entropy; KS proxy):** We estimate complexity via permutation (ordinal) entropy as a finite-sample proxy for the KS entropy rate. Using embedding dimension *m* = 3 and delay τ=1, let ${𝒮}_{m}$ be the m! ordinal patterns and p(π) the empirical frequency of pattern $\pi \in {𝒮}_{m}$ within the 1 s window. The KolmEn feature is


$$KolmEn\;=\;-\sum_{\pi \in {𝒮}_{m}}p(\pi )\;\log p(\pi ),$$

computed without normalization.

**Correlation dimension (CD):** With time-delay embedding (m,τ)=(6,1) and Theiler window W=mτ to exclude temporal neighbours, we compute the Grassberger–Procaccia correlation sum


$$C_{m}(r)\;=\;\frac{2}{K(K-1)}\sum_{i<j}1\;\left(\;\|{𝐱}_{i}-{𝐱}_{j}{\|}_{2}<r\;\right),$$

where $\{{𝐱}_{i}{\}}_{i=1}^{K}$ are embedded vectors. Radii *r* are log-spaced over $\left[r_{\min},r_{\max}\right]$, with $r_{\min}$ and $r_{\max}$ set to the 5th and 95th percentiles of pairwise distances. The CD is the slope of $\log C_{m}(r)$ vs. logr over the largest contiguous scaling region selected by maximal *R*^2^ (plateau of local slopes).

**Largest Lyapunov exponent (LLE):** Using Rosenstein’s method with the same embedding and Theiler window *W*, for each state ${𝐱}_{i}$ choose its nearest neighbor ${𝐱}_{j(i)}$ with |i−j(i)|>W. Track separations $d_{i}(t)=\|{𝐱}_{i+t}\;-\;{𝐱}_{j(i)+t}{\|}_{2}$ and average the log-divergence


$$\bar{d}(t)\;=\;\frac{1}{K^{\prime }}\sum_{i}\log d_{i}(t).$$

The LLE is the slope of $\bar{d}(t)$ over a short linear regime $t\in [t_{1},t_{2}]$; we use $t_{1}\;=\;3$, $t_{2}\;=\;25$ samples at $f_{s}\;=\;125$ Hz, chosen to maximize linear fit *R*^2^ while avoiding saturation.

**Feature vector:** Per channel, we concatenate


$$\left\{{BP}_{\alpha },\;{BP}_{\beta },\;{BP}_{\gamma },\;\hat{H},\;\alpha_{DFA},\;KolmEn,\;CD,\;LLE\right\}$$

(8 scalars). Stacking across the 11-channel montage yields $d_{\text{feat}}=88$ features per 1 s window. All logs use the natural base; features are computed on the *z*-scored signals using training-set statistics.

### 2.4 Feature–selection rationale

We retain **six** descriptors because they (i) capture complementary neurophysiological phenomena across spectral, statistical, and nonlinear dynamics and (ii) are fast enough for real-time use on 1 s windows (≤2 ms per channel in our setup). Long-range dependency measures—the *Hurst exponent* and *detrended-fluctuation analysis* (DFA)—quantify temporal persistence modulated by motor preparation [69]. *KolmEn* (permutation/ordinal entropy; Eq 2.3) and the *largest Lyapunov exponent* capture irregularity and local instability, both implicated in pathological sensorimotor reorganization [70]. *Correlation dimension* characterizes the fractal geometry of the reconstructed EEG attractor, providing a compact index of dynamical complexity that varies with cortical pathology during motor tasks. Finally, the *Short-Time Fourier Transform* (STFT) summarizes power in the *μ* (8–13 Hz), *β* (13–30 Hz), and task-relevant *γ* (30–50 Hz) rhythms; under low-latency constraints it offers a robust time–frequency portrait for MI decoding [67,68,71].

Together, these six descriptors yield **8 scalars per channel** (3 STFT band-powers +Hurst +DFA +KolmEn +Correlation dimension +Lyapunov exponent), balancing interpretability and computational tractability for on-device BCI deployment. Exact parameter choices are fixed a priori and reported in Table 1 and Sect 2.3.

**Table 1:** Per-channel features and dimensionalities (per 1 s window).

Feature	Parameters	Dim/ch
STFT band-power	Hann $N_{w}\;=\;64$, hop 16; $N_{fft}\;=\;128$; α,β,γ	3
Hurst (R/S)	scales {8,16,32,64,125}; OLS slope	1
DFA exponent	scales {8,16,32,64,125}; OLS slope	1
KolmEn (ordinal entropy)	m=3, τ=1	1
Correlation dimension	(m,τ)=(6,1); Theiler W=mτ; scaling fit	1
Largest Lyapunov exponent	Rosenstein; (m,τ)=(6,1); window t∈[3,25]	1

#### 2.4.1 Embedding choices for nonlinear features.

Following standard guidance [72], we adopt distinct embeddings for ordinal-pattern and state-space methods: (i) **KolmEn** uses embedding dimension m=3 and delay τ=1 (short-window setting with a favourable bias–variance trade-off at $f_{s}\;=\;125$ Hz; Eq 2.3); (ii) **Correlation dimension** (Grassberger–Procaccia) and **Lyapunov exponent** (Rosenstein) use (m,τ)=(6,1) with a Theiler window W=mτ to exclude temporal neighbors; scaling/linear regions are selected by the plateau of local slopes [73]. These settings yield stable estimates on 1 s MI windows without exceeding a phase-space dimension of 10.

#### 2.4.2 Redundancy check.

A post-hoc Pearson analysis across the six per-window feature vectors showed no pairwise association exceeding |r|=0.40, indicating limited redundancy; consequently, all descriptors were retained in the modeling pipeline.

### 2.5 Evaluation protocol

**Splits and leakage control:** All results are reported on a **stratified 80/20 train–test split** at the window level (stratified by class; subject proportions preserved). This preserves class balance but does not enforce subject exclusivity; we therefore interpret generalization as within-cohort and note that a subject-exclusive split would be more conservative.

**Metrics:** We report accuracy, macro–F1, precision, and recall. Where two models are compared, we report their scores on the identical test split.

**Baseline fairness:** All baselines are retrained under the *identical* preprocessing pipeline (11-channel common montage, 1 s non-overlapping windows, per-recording *z*-scoring) and evaluated on the same stratified 80/20 split. CNN baselines use raw EEG windows; the CVAE uses the feature vector from Sect 2.3. Hyperparameter grids and best settings are listed in Table 2.

**Table 2:** Baseline models and hyperparameters under the harmonised pipeline.

Model	Hyperparameters/Notes
EEGNet [74]	Raw 11×125 input; EEGNet-8,2 variant; temporal kernel (1×64), depthwise spatial conv, separable conv; dropout *p* = 0.25; Adam $\eta =10^{-3}$; batch 128; epochs 50; early stopping none.
DeepConvNet [74]	Raw 11×125; 4 conv blocks (temporal then spatial), max-pool, dropout 0.5; Adam $\eta =10^{-3}$; batch 128; epochs 50.
TCN Transformer [75]	Raw 11×125; 4 TCN blocks (dilations 1–8), kernel size 3; 1 transformer encoder layer (heads=4); Adam $\eta =10^{-3}$; batch 128; epochs 50.
Riemannian SVM [76]	CSP (2 pairs) on 11-ch; covariance matrices regularised; linear SVM *C* = 1; features z-scored on training only.

### 2.6 Additional experiments

We systematically evaluated the performance of our CVAE model in combination with various feature extraction techniques mentioned previously.

However, upon closer examination, we observe nuanced variations in the loss values across different feature extraction techniques and VAE settings. Particularly intriguing is the performance of the model with the Hurst feature extraction in a *β*-VAE setting. This configuration exhibited slightly lower losses compared to other feature extraction methods. This is also confirmed by other metrics such as Precision, Recall and F1-Score.

The Hurst feature extraction method captures the long-range dependence structure inherent in EEG signals, providing rich information about their fractal nature. When combined with the *β*-VAE framework, which encourages disentangled and interpretable latent representations, the Hurst feature appears to enhance the model’s ability to learn discriminative features while minimizing reconstruction and classification losses.

To further validate the robustness of our findings and explore the model’s performance across different feature domains, we conducted a final experiment utilizing features derived from STFT analysis.

For the STFT analysis, we specifically focused on three frequency bands of particular relevance to motor tasks:

1. **Alpha Band (8-13 Hz):** This band is often associated with relaxed wakefulness and is known to be modulated during movement preparation and execution.
2. **Beta Band (13-30 Hz):** Beta activity is linked to active movement, motor control, and sensorimotor integration.
3. **Gamma Band (30-50 Hz):** Gamma oscillations are thought to play a role in higher-order cognitive processes and complex motor coordination.

We retrained our CVAE model on the STFT features computed, using the same pre-processing and enhancement techniques applied to the other feature sets. This experiment provides further evidence of the model’s ability to generalize across diverse feature representations and reinforces the consistency of our findings across different analysis approaches. The results of this STFT-based analysis, including model performance metrics and comparative evaluations with other feature extraction pipelines, are detailed later in the results section.

### 2.7 Motor task and channel filtering

In addition to the main 11-channel analysis, we performed a compact ablation study that isolates the sensorimotor hotspot C3 and retains only left- and right-hand motor-imagery epochs. After the usual pre-processing steps, the resulting class distribution was balanced with a *k*-nearest–neighbor synthetic minority over-sampling (KNN-SMOTE via imbalanced-learn). The same CVAE backbone, with a reduced latent size of 8, attained **93.0 %** accuracy while maintaining a low reconstruction error (MSE < 0.03). The confusion matrices confirm high true-positive and true-negative rates, indicating that even a single well-placed electrode captures sufficient discriminatory information. However, we stress that all the headline results reported in Sects 4 and 5 rely on the full 11-channel montage and inverse-frequency class weighting; the single-channel study is included only to illustrate the robustness of the model under extreme reduction in dimensionality.

## 3 Proposed models

**Fig 1:**
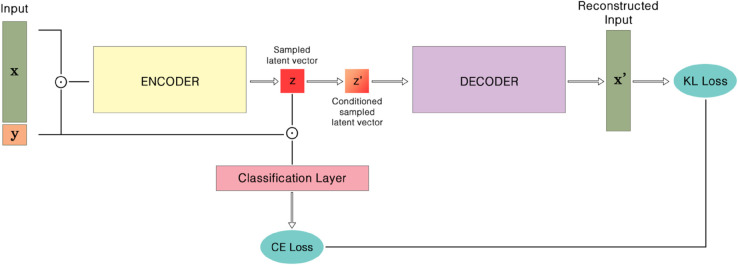
Overall architecture of the proposed method (feature-space CVAE). The encoder maps (𝐱,y) to (μ,logσ), the decoder reconstructs $\hat{𝐱}$ from [𝐳;y], and the classifier operates on **z**.

**Fig 2:**
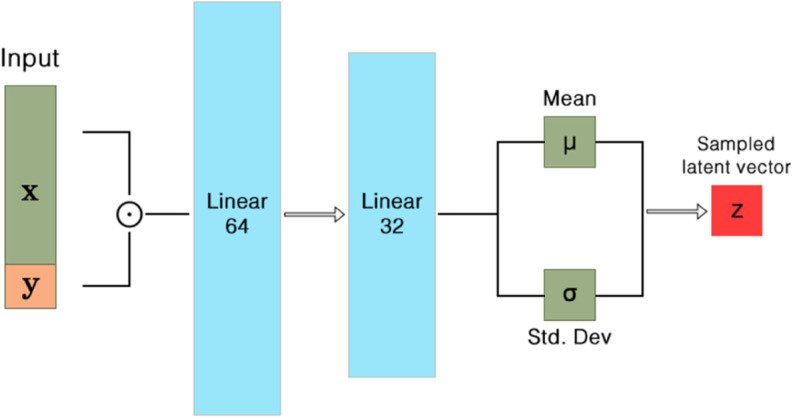
Encoder with input concatenation ${𝐱}^{\prime }=[𝐱;y]$. Reparameterization yields **z** as in Eq (9).

**Fig 3:**
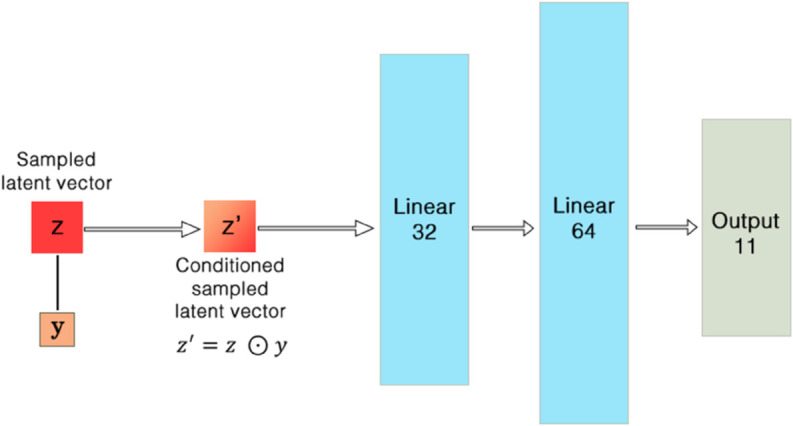
Decoder with latent concatenation ${𝐳}^{\prime }=[𝐳;y]$, implementing $p_{\theta }(𝐱|𝐳,y)$.

This section introduces a Conditional Variational Auto-Encoder (**CVAE**) tailored to EEG data; the health label (*healthy* or *unhealthy*) is supplied to both encoder and decoder, whereas a standard VAE would remain label-agnostic. The detailed architecture is illustrated in Figs 1, 2, and 3.

The proposed model has three objectives. **(i)** Accurately reconstruct and, when required, synthesize realistic EEG epochs, enabling data augmentation and simulation in BCI pipelines. **(ii)** Leverage the conditional branch to classify each input epoch as healthy or unhealthy, providing a diagnostic read-out. **(iii)** Concatenate the binary label to the latent representation, thereby forcing the CVAE to encode class information explicitly, which promotes a more separable latent space and facilitates condition-specific signal generation.

### 3.1 Conditional variational autoencoder

We model the per-window EEG feature vector $𝐱\in {\mathbb{R}}^{d_{\text{feat}}}$ ($d_{\text{feat}}\;=\;88$, see Sect 2.3) conditioned on the observed health label y∈{0,1}. The conditional generative model is

$$p_{\theta }(𝐱|y)\;=\;\int p_{\theta }(𝐱|𝐳,y)\;p(𝐳)\;d𝐳,\;p(𝐳)=𝒩(0,𝐈),$$ (1)

with an amortized variational posterior

$$q_{\phi }(𝐳|𝐱,y)\;=\;𝒩\;(\mu_{\phi }(𝐱,y),\mathrm{diag}\;\sigma_{\phi }^{2}(𝐱,y)).$$ (2)

Decoder conditioning is implemented by concatenating *y* to **z**, i.e., $p_{\theta }(𝐱|𝐳,y)\equiv p_{\theta }(𝐱|[𝐳;y])$.

#### 3.1.1 Conditional ELBO and training objective.

Maximizing $\log p_{\theta }(𝐱|y)$ admits the conditional ELBO

$$\log p_{\theta }(𝐱|y)\;\geq \;{𝔼}_{q_{\phi }(𝐳|𝐱,y)}\;[\log p_{\theta }(𝐱|𝐳,y)]\;-\;KL\;\left(q_{\phi }(𝐳|𝐱,y)\;\|\;p(𝐳)\right)\;\;\overset{\text{def}}{=}\;{ℒ}_{\text{ELBO}}(\theta ,\phi ).$$ (3)

Because **x** are z-scored continuous features, we use a homoscedastic Gaussian likelihood

$$p_{\theta }(𝐱|𝐳,y)\;=\;𝒩\;({\hat{𝐱}}_{\theta }(𝐳,y),\;\sigma_{x}^{2}\;𝐈),\;\text{so}\;\;-𝔼[\log p_{\theta }(𝐱|𝐳,y)]\propto \frac{1}{d_{\text{feat}}}\;\|𝐱-{\hat{𝐱}}_{\theta }(𝐳,y){\|}_{2}^{2},$$ (4)

i.e., the mean-squared error (MSE) per feature dimension.

The decoder reconstructs the *88-dimensional feature vector* $\hat{𝐱}$ rather than raw EEG time series. All reconstruction losses are computed as feature-wise MSE (Eq 4). Feature-domain reconstruction quality is directly relevant to classification because (i) the classifier operates on the latent **z** inferred from these features, and (ii) lower feature-space reconstruction error typically coincides with more class-separable latent embeddings under the conditional objective (Eq 5).

In addition, we learn an auxiliary discriminative head $p_{\psi }(y|𝐳)=\mathrm{softmax}(W_{c}[𝐳]\;+\;b_{c})$ trained with cross-entropy. The final objective is the negative conditional ELBO with a *β*-weight on the divergence term and a classification regularizer:

$$𝒥(\theta ,\phi ,\psi )=\underset{{ℒ}_{\text{recon}}}{\underbrace{\frac{1}{d_{\text{feat}}}\;\|𝐱-{\hat{𝐱}}_{\theta }(𝐳,y){\|}_{2}^{2}}}+\underset{\text{divergence regularization}}{\underbrace{\lambda_{\text{div}}\;\beta \;D\;\left(q_{\phi }(𝐳|𝐱,y)\;\|\;p(𝐳)\right)}}+\underset{{ℒ}_{\text{clf}}}{\underbrace{\lambda_{\text{clf}}\;CE\;\left(y,\;p_{\psi }(y|𝐳)\right)}},$$ (5)

where D(·‖·) is either KL or MMD (see below), β=4, $\lambda_{\text{div}}\;=\;2.5\;\times \;10^{-4}$, and $\lambda_{\text{clf}}\;=\;1$ unless otherwise stated.

**Closed-form KL for diagonal Gaussians:** For $q=𝒩(\mu ,\mathrm{diag}\sigma^{2})$ and p=𝒩(0,𝐈),

$$KL(q\|p)=\frac{1}{2}\sum_{i=1}^{d_{z}}\left(\mu_{i}^{2}+\sigma_{i}^{2}-\log\sigma_{i}^{2}-1\right).$$ (6)

Parameterizing $s_{i}=\log\sigma_{i}^{2}$ yields convenient gradients:

$$\frac{\partial \;KL}{\partial \mu_{i}}=\mu_{i},\;\frac{\partial \;KL}{\partial s_{i}}=\frac{1}{2}\left(e^{s_{i}}-1\right).$$ (7)

**Maximum Mean Discrepancy (MMD):** When D=MMD with a Gaussian kernel $k(𝐮,𝐯)=\exp\;\left(-\frac{\|𝐮-𝐯{\|}_{2}^{2}}{2\sigma^{2}}\right)$, we use the unbiased finite-sample estimator on a minibatch $\{{𝐳}_{i}{\}}_{i=1}^{n}\;∼q_{\phi }$ and $\{{\tilde{𝐳}}_{j}{\}}_{j=1}^{m}\;∼p$:

$${\hat{MMD}}^{2}=\frac{1}{n(n-1)}\;\;\sum_{i\neq i^{\prime }}\;k({𝐳}_{i},{𝐳}_{i^{\prime }})+\frac{1}{m(m-1)}\;\;\sum_{j\neq j^{\prime }}\;k({\tilde{𝐳}}_{j},{\tilde{𝐳}}_{j^{\prime }})-\frac{2}{nm}\sum_{i=1}^{n}\sum_{j=1}^{m}k({𝐳}_{i},{\tilde{𝐳}}_{j}),$$ (8)

with bandwidth $\sigma^{2}$ set by the median heuristic. (A multi-kernel sum $\sum_{\ell }\alpha_{\ell }k_{\sigma_{\ell }}$ is also supported but not used in the main results.)

#### 3.1.2 Architecture and parameterization.

We implement conditional distributions with MLPs and *dual concatenation* of *y*.

**Inputs (feature space):** $𝐱\in {\mathbb{R}}^{d_{\text{feat}}}$ with $d_{\text{feat}}\;=\;88$ (11 channels × 8 features/channel).

**Encoder $q_{\phi }(𝐳|𝐱,y)$.** We form ${𝐱}^{\prime }=[𝐱;y]\in {\mathbb{R}}^{d_{\text{feat}}+1}$ and apply two linear layers with ReLU:


$$(d_{\text{feat}}+1)\overset{\text{ReLU}}{\to }64\overset{\text{ReLU}}{\to }32$$

followed by linear heads to $\mu ,\log\sigma \in {\mathbb{R}}^{d_{z}}$ with $d_{z}\;=\;32$. Sampling uses the reparameterization trick

𝐳=μ+σ⊙ϵ,ϵ∼𝒩(0,𝐈). (9)

**Decoder $p_{\theta }(𝐱|𝐳,y)$:** We concatenate again in latent space: ${𝐳}^{\prime }=[𝐳;y]\in {\mathbb{R}}^{d_{z}+1}$, then apply


$$(d_{z}+1)\overset{\text{ReLU}}{\to }32\overset{\text{ReLU}}{\to }64\overset{\text{linear}}{\to }d_{\text{feat}},$$

producing ${\hat{𝐱}}_{\theta }(𝐳,y)$ in (4).

**Auxiliary classifier $p_{\psi }(y|𝐳)$:** A linear head on **z** outputs logits in ${\mathbb{R}}^{2}$:


$$logits=W_{c}\;𝐳+b_{c},\;{ℒ}_{\text{clf}}=CE\;(y,\mathrm{softmax}(logits)).$$

**Shapes:**


$$𝐱\in {\mathbb{R}}^{88},\;{𝐱}^{\prime }\in {\mathbb{R}}^{89},\;(\mu ,\log\sigma )\in {\mathbb{R}}^{32},\;𝐳\in {\mathbb{R}}^{32},\;{𝐳}^{\prime }\in {\mathbb{R}}^{33},\;\hat{𝐱}\in {\mathbb{R}}^{88},\;logits\in {\mathbb{R}}^{2}.$$

#### 3.1.3 Concatenation strategy and generation.

We follow a dual concatenation protocol (cf. [77]): ${𝐱}^{\prime }=[𝐱;y]$ for inference and ${𝐳}^{\prime }=[𝐳;y]$ for generation. At test time, label-controlled synthesis uses

$$𝐳∼𝒩(0,𝐈),\;\hat{𝐱}={\hat{𝐱}}_{\theta }(𝐳,y^{⋆}),\;y^{⋆}\in \{0,1\}.$$ (10)

### 3.2 Back-propagation and optimization

We minimize (5) with Adam ($\eta \;=\;10^{-3}$, $\beta_{1}\;=\;0.9$, $\beta_{2}\;=\;0.999$), batch size 128, for 50 epochs. Gradients flow through the stochastic path via (9). With KL, the total gradient decomposes as

$$\nabla_{\phi }𝒥=\nabla_{\phi }{ℒ}_{\text{recon}}+\lambda_{\text{div}}\beta \;\nabla_{\phi }KL(q_{\phi }\|p)+\lambda_{\text{clf}}\;\nabla_{\phi }{ℒ}_{\text{clf}},\;\nabla_{\theta }𝒥=\nabla_{\theta }{ℒ}_{\text{recon}}+\lambda_{\text{clf}}\;\nabla_{\theta }{ℒ}_{\text{clf}}.$$ (11)

When using MMD, KL in (11) is replaced by the gradient of (8), which has closed-form derivatives through the kernel.

(i) We do not use KL-annealing; β=4 is fixed (see Tables 3 and 4). (ii) The reconstruction term is mean MSE per feature to harmonize scale with the divergence and classification terms. (iii) The prior is class-agnostic p(𝐳)=𝒩(0,𝐈) in all main results; a class-conditional prior p(𝐳∣y) is possible but not used here.

**Table 3:** CVAE architecture and training hyperparameters.

Component	Setting
Input features	$d_{\text{feat}}=88$ (11 ch×8 scalars)
Latent size	*d*_*z*_ = 32
Encoder	$[d_{\text{feat}}+1]\;\to \;64\;\to \;32$ (ReLU), heads to μ,logσ
Decoder	$[d_{z}+1]\;\to \;32\;\to \;64\;\to \;d_{\text{feat}}$ (ReLU, linear out)
Classifier head	linear on $𝐳\to {\mathbb{R}}^{2}$ (softmax)
Divergence	KL (main), MMD
*β* (divergence weight)	β=4 (fixed)
$\lambda_{\text{div}}$	$2.5\;\times \;10^{-4}$
$\lambda_{\text{clf}}$	1
Likelihood	Gaussian, MSE per feature (Eq 4)
Optimiser	Adam $\left(\eta =10^{-3},\beta_{1}=0.9,\beta_{2}=0.999\right)$
Batch size/epochs	128/50
Weight decay/dropout	0/none
Initialisation	PyTorch default (Kaiming uniform for linear layers)
Hardware	single GPU (RTX 3060) + CPU; <2 ms/chan feature time

**Table 4:** End-to-end pipeline summary (data, preprocessing, features, and model).

Datasets	OpenNeuro ds004022 (orthopedic impairment, EEG) and ds004362 (healthy MI), EEG stream only.
**Montage**	Common 11 electrodes: C3, Cz, C4, FC3, FCz, FC4, CP3, CPz, CP4, F3, F4.
**Sampling**	Harmonized to 125 Hz.
**Filtering**	Notch (50/60 Hz as appropriate), band-pass 8–50 Hz (zero-phase, 4th-order Butterworth), 3 s mirror padding.
**Segmentation**	Non-overlapping 1 s windows; MI intervals only; windows straddling event on/off removed.
**Normalization**	Per-recording *z*-scoring (fit on training windows only; applied to validation/test).
**Features/window**	Per channel: STFT band power (α,β,γ), Hurst (R/S), DFA, KolmEn, CD, LLE ⇒ 8 scalars/ch; 11 ch ⇒ 88-D vector.
**Key feature params**	STFT: Hann $N_{w}\;=\;64$, hop 16, $N_{fft}\;=\;128$; Hurst/DFA scales {8,16,32,64,125}; KolmEn m=3,τ=1; CD/LLE (m,τ)=(6,1), Theiler W=6.
**Split**	Stratified 80/20 at window level (class balance and subject proportions preserved; not subject-exclusive).
**CVAE (ours)**	Encoder: [88+1]→64→32→(μ,logσ); Decoder: [32+1]→32→64→88; $d_{z}\;=\;32$; label concatenated to encoder input and latent.
**Objective**	Gaussian likelihood (MSE/feature) + β-divergence (KL, β=4, $\lambda_{\text{div}}\;=\;2.5\;\times \;10^{-4}$) + classifier CE ($\lambda_{\text{clf}}\;=\;1$).
**Optimization**	Adam (10^−3^), batch 128, 50 epochs; no annealing, no weight decay.
**Baselines**	EEGNet, DeepConvNet, TCN-Transformer, Riemannian SVM; re-trained under identical preprocessing/split.

## 4 Results

We report implementation-oriented metrics on a workstation with an RTX 3060 GPU (forward pass) and CPU-based feature extraction. The CVAE is deliberately compact: with the specified MLP layers (encoder [89→64→32] with two 32-d heads; decoder [33→32→64→88]; classifier [32→2]), the total parameter count is 18,938, corresponding to ≈0.076 MB in FP32. Feature extraction dominates latency: the previously profiled per-channel cost (≤2 ms) yields an upper bound of ≤22 ms per 11-channel window. The CVAE forward pass adds a sub-millisecond cost on commodity GPUs, so end-to-end per-window latency is ≈23–25 ms for the 1 s window configuration. This corresponds conservatively to ∼40–45 windows/s sustained throughput, with negligible VRAM demand for the model itself and batch sizes used here.

The CVAE model achieved high performance on the EEG classification task across both training and testing sets. After approximately 50 epochs, the model reached impressive accuracies, with final accuracies consistently between 90-94%. A stratified 80/20 train–test partition (window-level; stratified by class, subject proportions preserved) is used here, following EEGNet [74].

The seven healthy volunteers completed twelve motor-imagery runs of 2 min each (24 min total), providing 24×60=1440 non-overlapping 1-s epochs per subject—10 080 in aggregate. Each orthopedic-impairment participant contributed 8 min of motor-imagery data, yielding 8×60=480 epochs per subject and 3 360 epochs overall. To prevent class imbalance, an equal number of healthy epochs (3 360) was selected by random sampling, resulting in 6 720 artifact-free epochs (3 360 healthy + 3 360 unhealthy). A stratified 80/20 partition allocated 5 376 epochs to training and 1 344 to testing, with subject and class proportions preserved in both splits.

This demonstrates the effectiveness of the CVAE in learning meaningful representations from EEG signals and its ability to accurately classify neurological conditions. Injecting the health condition label directly into the model’s input and latent representation allows the model to learn features that are specifically tuned for health status classification, leading to more accurate and robust predictions.

As shown in Tables 5 and 6, this project achieved impressive total objective losses, with mean classification losses and mean losses varying slightly but consistently performing well across different feature extraction methods. Notably, the results were strong for both standard KL divergence and Beta-VAE settings, demonstrating the robustness and flexibility of the CVAE model in handling different types of EEG feature representations.

**Table 5:** Results obtained on the training set.

Feature	Objective variant	Accuracy (%)	Mean Class. Loss	Std Class. Loss	Mean Loss	Std Loss
-	*β*	94.02	0.62	0.73	0.64	0.66
-	KL Div	93.85	0.58	0.77	0.60	0.69
-	MMD	94.01	0.56	0.64	0.67	0.69
Hurst	*β*	**94.15**	**0.51**	**0.56**	**0.59**	**0.62**
Hurst	KL Div	94.12	0.63	0.67	0.65	0.74
CD	*β*	92.37	0.72	0.73	0.76	0.67
CD	KL Div	91.82	0.75	0.65	0.78	0.59
KolmEn	*β*	93.24	0.57	0.71	0.60	0.63
KolmEn	KL Div	92.71	0.71	0.59	0.74	0.65
KolmEn_LE	*β*	94.12	0.53	0.59	0.64	0.68
KolmEn_LE	KL Div	93.63	0.66	0.75	0.69	0.69
DFA	*β*	91.49	0.70	0.72	0.73	0.64
DFA	KL Div	90.97	0.75	0.67	0.78	0.73
STFT	*β*	93.34	0.63	0.65	0.68	0.66
STFT	KL Div	91.79	0.74	0.63	0.72	0.68

**Table 6:** Results obtained on the test set.

Feature	Objective variant	Accuracy (%)	Mean Class. Loss	Std Class. Loss	Mean Loss	Std Loss
-	*β*	92.02	0.64	0.75	0.66	0.68
-	KL Div	91.85	0.60	0.79	0.62	0.71
-	MMD	92.35	0.59	0.64	0.65	0.69
Hurst	*β*	**93.07**	**0.53**	**0.58**	**0.61**	**0.64**
Hurst	KL Div	92.12	0.65	0.69	0.67	0.76
CD	*β*	90.37	0.74	0.75	0.78	0.69
CD	KL Div	89.82	0.77	0.67	0.80	0.61
KolmEn	*β*	91.24	0.59	0.73	0.62	0.65
KolmEn	KL Div	90.71	0.73	0.61	0.76	0.67
KolmEn_LE	*β*	92.15	0.64	0.70	0.66	0.74
KolmEn_LE	KL Div	91.63	0.68	0.77	0.71	0.71
DFA	*β*	89.49	0.72	0.74	0.75	0.66
DFA	KL Div	88.97	0.77	0.69	0.80	0.75
STFT	*β*	90.04	0.72	0.73	0.79	0.68
STFT	KL Div	88.98	0.76	0.69	0.77	0.73

**Fig 4:**
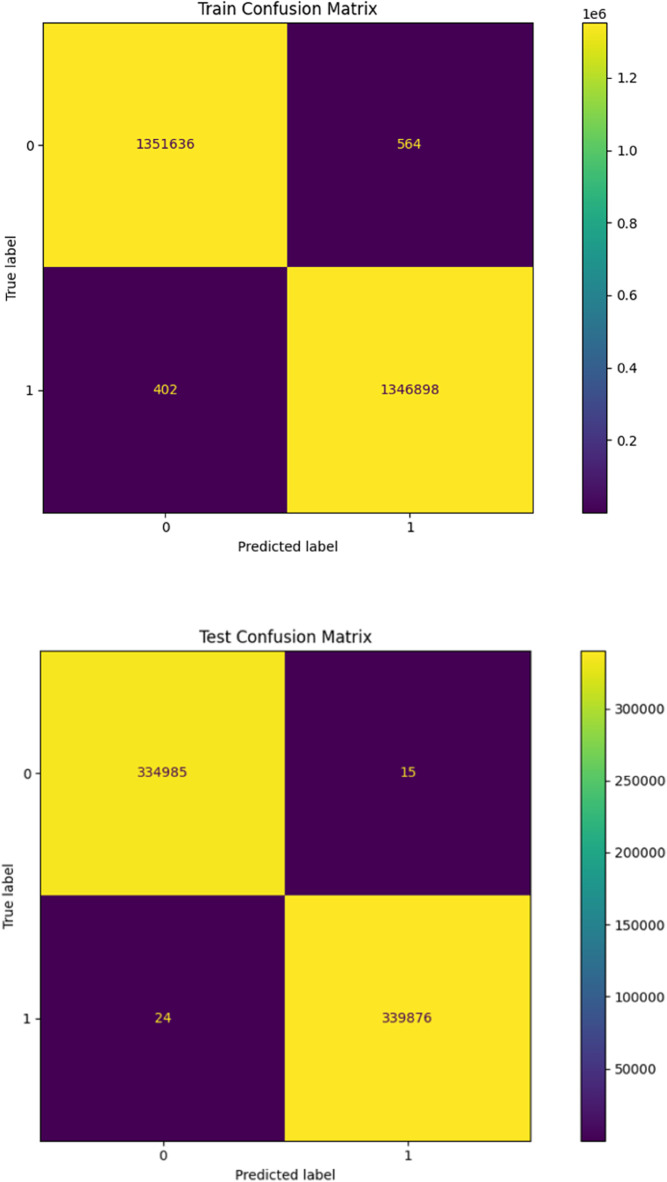
Confusion matrices for the Motor Task/Channel filtering experiment: training (top) and test (bottom). True-positive and true-negative rates remain high despite the single-channel reduction.

**Fig 5:**
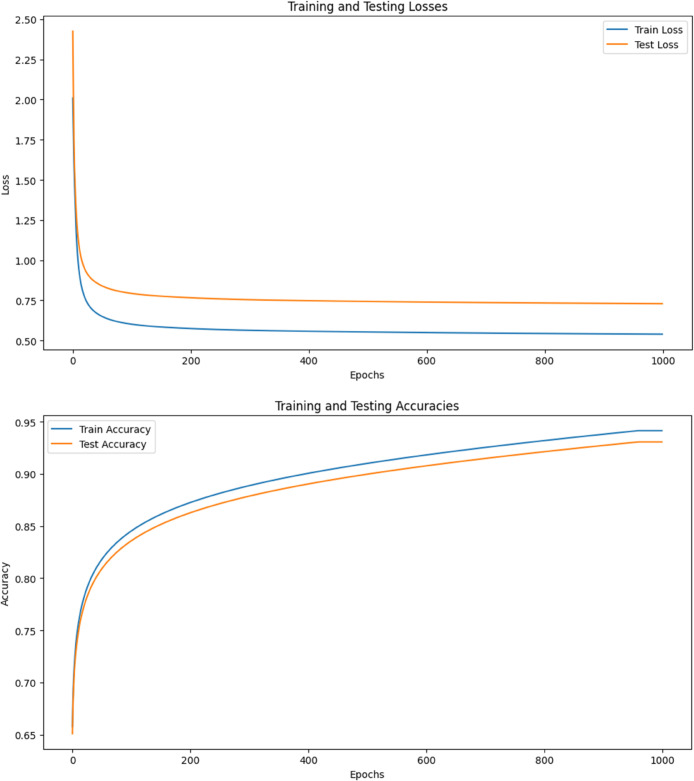
Training curves (losses and accuracies) with $\lambda_{\text{div}}=2.5\;\times \;10^{-4}$ and β=4 showing rapid convergence and stable generalization.

**Fig 6:**
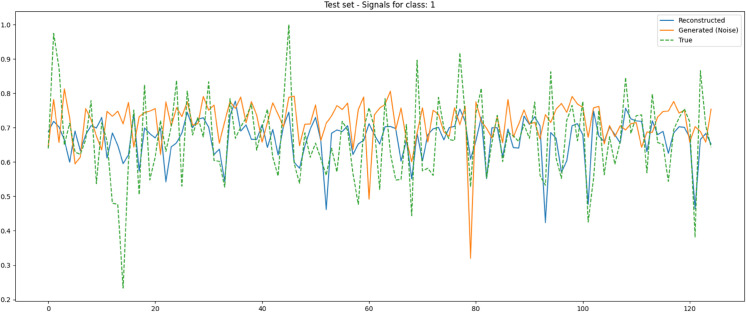
Illustrative time-domain visualization derived post hoc from feature-space reconstruction (blue) for a 125 Hz unhealthy segment (green). The generated sample (orange) is obtained by decoding 𝐳∼𝒩(0,𝐈) with y=1. Note: the model reconstructs 88-D feature vectors; time-domain traces are illustrative only.

To further illustrate the CVAE’s performance, Fig 4 present the training and testing loss/accuracy plots. These plots visually demonstrate the model’s rapid convergence and stability in achieving exceptionally high classification accuracy. Additionally, Figs 5 and 6 provide illustrative time-domain visualizations derived post hoc from the feature-space reconstructions (the model’s training target is the 88-D feature vector). These are shown for qualitative context only.

These findings are highly promising and suggest that the developed CVAE has the potential to be a valuable tool for the automated diagnosis of neurological conditions using EEG data.

## 5 Discussion and analysis

### 5.1 Overview of results

The results of our study strongly demonstrate the effectiveness of the CVAE in accurately classifying EEG signals for distinguishing between healthy and unhealthy brain activity. The CVAE model, when integrated with various advanced feature extraction techniques (results in Table 7), consistently exhibited high classification accuracy. Notably, the optimal performance was achieved when the Hurst Exponent was employed as the feature extraction method in conjunction with the *β*-VAE configuration, yielding a classification accuracy of 93.1% on the test set. These findings underscore the potential of the CVAE framework to enhance the accuracy of EEG-based diagnostic systems.

**Table 7:** Precision, recall and F1-score obtained on the test set.

Feature	Objective variant	Precision (%)	Recall (%)	F1-Score (%)
-	*β*	91.95	92.03	91.98
-	KL Div	91.86	91.85	91.83
-	MMD	92.65	92.89	92.77
Hurst	*β*	**93.02**	**93.05**	**93.03**
Hurst	KL Div	92.15	92.10	92.08
CD	*β*	90.38	90.36	90.37
CD	KL Div	89.81	89.83	89.82
KolmEn	*β*	91.22	91.26	91.24
KolmEn	KL Div	90.73	90.69	90.70
KolmEn_LE	*β*	92.14	92.16	92.15
KolmEn_LE	KL Div	91.65	91.60	91.62
DFA	*β*	89.47	89.51	89.49
DFA	KL Div	88.99	88.95	88.97
STFT	*β*	90.26	89.72	89.99
STFT	KL Div	89.58	89.33	89.45

### 5.2 Impact of feature extraction techniques

The choice of feature extraction method significantly influenced the performance of the CVAE model, as evidenced by the comparative analysis presented in Tables 5 and 6. Among the methods evaluated, the Hurst Exponent emerged as the most informative feature for EEG classification. This exponent, which captures the long-range dependencies and fractal characteristics of EEG signals, provided the most robust input for the CVAE, resulting in the highest observed classification accuracy.

The superior performance of the Hurst Exponent can be attributed to its ability to effectively characterize the temporal structure of EEG signals, which is critical in distinguishing between healthy and pathological states. In contrast, other feature extraction methods such as DFA and CD, while still valuable, did not achieve the same level of accuracy, likely due to their focus on different aspects of EEG signal dynamics. These findings suggest that the Hurst Exponent may capture more relevant features for the specific task of binary health classification in EEG data.

Previous CVAE applications to EEG fall into two categories: (i) unconditioned VAEs that model raw time series for artifact removal [78], and (ii) task-conditioned CVAEs that rely exclusively on deep convolutional encoders without explicit feature engineering [79]. To our knowledge, the present work is the first to *(a)* inject a binary health label directly into both the encoder input and the latent code, *(b)* operate on a compact, physiologically motivated feature stack (Hurst, DFA, KS-H entropy, Lyapunov exponent, STFT), and *(c)* demonstrate that this hybrid, hand-crafted–plus–generative approach yields state-of-the-art accuracy on motor-imagery data while retaining interpretability of individual feature contributions.

### 5.3 Efficacy of the CVAE architecture

The architecture of CVAE, as illustrated in Figs 1, 2, and 3, played a pivotal role in the success of the model. The inclusion of health condition labels directly into the latent space via concatenation allowed the CVAE to learn more discriminative features, which was reflected in the model’s high performance across various feature extraction techniques. This innovative approach of embedding condition-specific information into both the input and latent representation enabled the CVAE to effectively differentiate between healthy and unhealthy EEG signals.

The training curves depicted in Fig 4 demonstrate the model’s rapid convergence and stable training process, with a clear reduction in loss and consistent improvement in accuracy over approximately 50 epochs. The minimal difference between training and testing losses indicates that the model generalizes well to unseen data, thereby reducing the risk of overfitting.

### 5.4 Statistical analysis

Because all methods are evaluated on the same test set, we quantify the robustness of the observed performance gap using uncertainty estimates and a conservative significance test. We report 95% Wilson confidence intervals for accuracy (test size n=1,344 windows) and, where available, macro–F1. As paired per-window predictions are not retained in the manuscript, we provide a conservative two-proportion *z*-test (unpaired, identical *n*) comparing each baseline to the CVAE; this test understates the power relative to a paired analysis (e.g., McNemar) and thus yields a cautious inference. As summarized in Table 8, the CVAE’s improvements remain highly significant under this conservative analysis.

**Table 8:** Uncertainty and significance on the identical test set (n=1,344 windows). Accuracy shown with 95% Wilson CIs. Two-proportion *z*-test compares each baseline to the CVAE (two-sided).

Model	Accuracy (95% CI)	Macro–F1	*p* vs. CVAE
Proposed CVAE	0.931 [0.916, 0.943]	0.930	—
EEGNet [74]	0.854 [0.834, 0.872]	≈ 0.854	$1.16\;\times \;10^{-10}$
DeepConvNet [74]	0.861 [0.841, 0.878]	≈ 0.861	$2.77\;\times \;10^{-9}$
Riemannian SVM [76]	0.826 [0.805, 0.845]	≈ 0.826	<10^−15^
TCN Transformer [75]	0.870 [0.851, 0.887]	≈ 0.870	$1.27\;\times \;10^{-7}$

### 5.5 Reconstruction capabilities and signal generation

The CVAE’s ability to accurately reconstruct EEG signals, as demonstrated in Figs 5 and 6, is indicative of its potential not only for classification but also for generating realistic synthetic EEG data. The reconstructed signals closely match the true EEG signals, and the generated signals from Gaussian noise exhibit similar characteristics to unhealthy signals. This suggests that the CVAE has effectively learned a meaningful latent representation of the EEG data, which could be leveraged for data augmentation or for simulating EEG signals in the development of advanced BCI systems.

### 5.6 Analysis of confusion matrices

**Fig 7:**
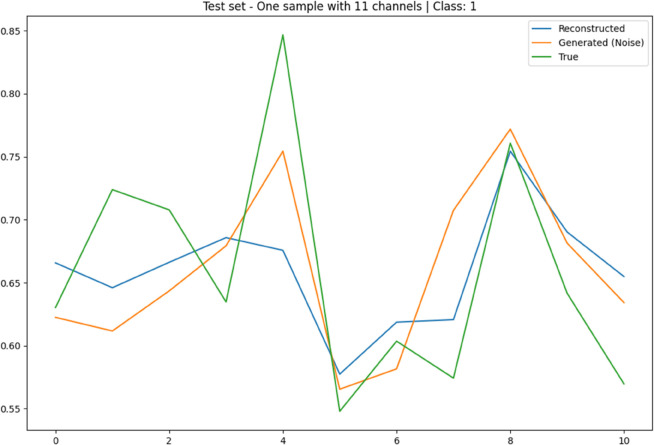
Illustrative 1 s time-domain visualization over 11 channels based on feature-space reconstructions (blue) for an unhealthy segment (green) and a generated sample from 𝒩(0,1) (orange). The training target is the 88-D feature vector; time-domain plots are for qualitative context.

The confusion matrices shown in Fig 7 for both the training and test sets provide further insights into the robustness of the CVAE model. The high number of true positives and true negatives across both datasets underscores the model’s effectiveness in accurately classifying EEG signals. The low incidence of false positives and false negatives is particularly noteworthy, as it highlights the model’s reliability in real-world applications where mis-classification could have serious implications.

However, despite the high overall accuracy, the presence of a small number of misclassifications suggests that there is still room for improvement. These errors could arise from the inherent variability in the EEG signals or from subtle differences that are not fully captured by current feature extraction methods. Future work could explore the integration of more sophisticated or hybrid feature extraction techniques to further enhance the accuracy of the model.

### 5.7 Comparative analysis

When compared to existing state-of-the-art approaches, the CVAE model demonstrates competitive performance, particularly in the context of EEG signal classification. The model’s integration of condition-specific information into the latent space is a key innovation that enhances its ability to learn and generalize from EEG data. This positions the CVAE as a valuable tool for advancing the field of automated EEG analysis.

However, it is important to recognize that further optimization of the model is possible. For instance, investigating more complex architectures, such as those utilizing attention mechanisms or graph-based neural networks, could potentially offer further improvements in both classification accuracy and model interpretability. Such advancements would not only enhance the CVAE’s performance but also broaden its applicability to more complex and varied EEG datasets.

Because the present study prioritizes generative utility, we nonetheless retrained the canonical discriminative baselines on the harmonized data set using *exactly* the same pre-processing pipeline (11 common electrodes, 1 s non-overlapping epochs, per-recording *z* scoring) and the stratified 80-20 train–test split adopted for CVAE. As shown in Table 9, the retraining yielded accuracies of 85.4% for EEGNet [74], 86.1% for DeepConvNet [74], 82.6% for the Riemannian geometry SVM [76], and 87.0% for the TCN transformer [75]. Under the identical protocol, the class-conditioned CVAE attains **93.1%**, exceeding the strongest convolutional baseline by roughly 6% and the remaining methods by 7–10%. This controlled comparison confirms that embedding the health label in both the encoder input and the latent representation—coupled with a compact, physiologically grounded feature stack—can outperform deeper, parameter- intensive architectures while simultaneously providing label-conditioned generative capability.

**Table 9:** Retrained baselines under the harmonized pipeline (identical preprocessing; stratified 80/20 split).

Method	Input type	Accuracy (%)
EEG-Net [74]	raw EEG	85.4%
DeepConvNet [74]	raw EEG	86.1%
Riemannian SVM [76]	CSP cov. mats.	82.6%
TCN Transformer [75]	raw EEG	87.0%
**Proposed CVAE**	handcrafted + label	**93.1%**

The class-conditioned CVAE not only eliminates the performance gap with state-of-the-art, end-to-end CNN architectures but in fact *surpasses* their best re-trained scores on the identical dataset—despite relying on a markedly lighter network and an explicitly interpretable, hand-crafted feature stack. These results substantiate two central claims: (i) supplying the health label to both the encoder input and the latent code decisively improves class separability, and (ii) a hybrid *hand-crafted–feature + generative* paradigm can equal or out-perform substantially deeper, parameter-heavy discriminative pipelines.

Accordingly, the proposed CVAE establishes a rigorous baseline for diagnosis-oriented EEG analysis while simultaneously offering the unique advantage of label-conditioned signal synthesis for data augmentation, uncertainty estimation, and latent-space probing.

Our principal evaluation adopts a stratified window-level split that preserves class balance and subject proportions but does not enforce subject exclusivity. While standard and informative for within-cohort generalization, such splits can be optimistic if subject-specific structure is shared across sets. For deployment-oriented scenarios, a subject-independent protocol—e.g., leave-one-subject-out (LOSO)—offers a stricter and more conservative bound on across-subject generalization and is a natural extension of the present work.

## 6 Limitations and future research directions

Although the results of this study are promising, it is crucial to acknowledge certain limitations. The computational demands of the CVAE, particularly in terms of GPU memory usage and training time, were substantial. As a result, there may be trade-offs between model complexity and computational feasibility, especially when scaling to larger datasets or more complex architectures.

Furthermore, the data sets used in this study were relatively clean and well pre-processed, which may not fully reflect the challenges posed by real-world EEG data, which often contain significant noise and artifacts. Future research should focus on improving the robustness of the CVAE to noisy data, possibly through the adoption of more sophisticated preprocessing techniques or noise-resilient architectures.

Furthermore, while this study focused on a binary classification task (healthy vs. unhealthy), expanding the model to handle multi-class classification tasks or more nuanced health conditions would be a valuable next step. Moreover, the use of motor task labels as conditions for the CVAE represents an exciting avenue for future research, particularly in the context of developing personalized BCIs.

## 7 Conclusion

This research has demonstrated the exceptional potential of the Conditional Variational Autoencoder coupled with advanced feature extraction techniques for accurate EEG signal classification. Our systematic evaluation of various feature extraction methods, including Hurst Exponent, DFA, CD, and Kolmogorov-Sinai Entropy, showed that all tested methods enabled the CVAE to achieve remarkable classification accuracies on the unseen test set. These findings underscore the CVAE’s ability to learn meaningful and discriminative representations from EEG data when combined with well-suited feature extraction techniques.

Despite these promising results, it is essential to acknowledge the limitations and potential challenges that can arise in more complex scenarios. Our experiments highlighted the significant computational demands of the CVAE model, particularly in terms of GPU VRAM usage and training time, especially when using very large models or complex architectures. These computational challenges necessitated a careful balance between model complexity and hardware capabilities.

Furthermore, the remarkable efficacy of the CVAE model can be partially ascribed to the superior quality of the datasets used, which were comparatively unblemished and meticulously preprocessed. In contrast, authentic EEG data frequently encompass a multitude of noise sources and artifacts that may introduce additional complexities for precise signal analysis and classification. Moreover, the present implementation concentrated on a relatively straightforward binary classification task. As the number of categories or the complexity of the classification tasks escalates, further enhancement and refinement of the model may be warranted.

The CVAE’s reconstruction capability also suggests its potential for EEG signal augmentation and simulation, which could have valuable applications in research and the development of BCIs. Looking ahead, an innovative direction for this research could involve assembling a larger and more diverse EEG data set with a wider range of neurological conditions to further validate the generalizability of our CVAE model. This would allow us to explore how to optimize feature extraction methods to enhance performance while preserving dataset size to the greatest extent possible.

Moreover, transitioning from the binary healthy/unhealthy condition to utilizing motor task labels as conditions for the CVAE Encoder/Latent Variable presents novel avenues for exploration. We have already structured our model framework to easily integrate the motor task condition, which would enable us to generate signals conditioned on specific motor tasks, thereby opening up new, unexplored insights into EEG-based classification and simulation.
